# ComWΔ6 Stimulates Transcription of Pneumococcal Competence Genes *in vitro*

**DOI:** 10.3389/fmolb.2020.00061

**Published:** 2020-05-06

**Authors:** Nicole L. Inniss, Donald A. Morrison

**Affiliations:** Department of Biological Sciences, The University of Illinois at Chicago, Chicago, IL, United States

**Keywords:** alternative sigma factor, *S. pneumoniae* competence, genetic transformation, ComW, transcription

## Abstract

The alternative streptococcal σ-factor and master competence regulator, σ^X^, stimulates transcription from competence promoters, *in vitro.* As the only known alternative σ-factor in streptococci, σ^X^ expression is tightly controlled in each species and has a specific physiological role. Pneumococcal transformation also requires the DNA binding activity of ComW, a known σ^X^ activator and stabilizer. Mutations to the housekeeping σ factor, σ^A^, partially alleviate the ComW requirement, suggesting that ComW is a key player in the σ factor swap during the pneumococcal competence response. However, there is no evidence of a direct ComW – RNA polymerase interaction. Furthermore, if and how ComW functions directly at combox promoters is still unknown. Here we report that a DNA-binding ComW variant, ComΔ6, can stimulate transcription from σ^X^ promoters *in vitro*.

## Introduction

Streptococci are Gram-positive commensal cocci for a wide range of animal hosts, including humans. *Streptococcus pneumoniae* (pneumococcus), an inhabitant of the human nasopharynx, is a causative agent of pneumonia and meningitis in children, the elderly, and immune-compromised individuals. Natural genetic transformation (NGT) was discovered in the pneumococcus (Griffith, 1928; Avery and MacLeod, 1944), and subsequently the genes required for transformation have been found in the genomes of streptococci from all species groups (Håvarstein, 2010). Their genomes encode transcriptional regulators and apparatuses that enable exogenous DNA uptake and genetic recombination. This ability yields highly diverse genomes, making streptococci well equipped for adaptation, as seen with frequent capsular switching and the rapid spread of genes that mediate antibiotic resistance (Andam and Hanage, 2015).

Pneumococci primed for transformation must develop competence, a transient state marked by a shift in both transcriptomic and proteomic profiles, including two waves of new gene transcription, yielding early and late competence gene groups (Peterson et al., 2000; Fontaine et al., 2010; Gao et al., 2014; Khan et al., 2016, 2017). The link between early gene expression and late gene expression is the production of the alternative σ-factor, SigX (σ^X^), a member of the σ^70^ family of proteins (Lee and Morrison, 1999, 2000; Luo and Morrison, 2003). All bacteria produce the principal σ factor σ^A^, responsible for most gene transcription, and many produce multiple alternative σ factors that promote specific cellular responses, however, σ^X^ is the only alternative σ factor produced in pneumococcus (Håvarstein, 2010), and *sigX* expression is strictly linked to competence development.

Streptococci utilize tightly regulated quorum-sensing systems to coordinate σ^X^- mediated competence, as well as other group behaviors (Cheng et al., 1997; Peterson et al., 2004; Mashburn-Warren et al., 2010, 2012). Species of the angionsus and mitis groups use the ComCDE pathway (Cheng et al., 1997), an auto-regulated two-component signal transduction system (TCSTS), that responds to the Competence Stimulating Peptide (CSP) (Håvarstein et al., 1995). Much of our understanding of streptococcal transformation comes from initial work done with the ComCDE system in pneumococcus.

During exponential growth, the σ^A^-RNA polymerase complex (σ^A^ holo-enzyme) basally transcribes *comC* leading to production of the 41 aa pro-peptide, ComC, 41 (Håvarstein et al., 1995). The ComC pro-peptide is simultaneously cleaved and exported to form mature CSP by the ABC-transporter, ComAB (*comAB*). Extracellular CSP is sensed by the histidine-kinase receptor, ComD, resulting in auto-phosphorylation and activation of its cognate response regulator, ComE, via a phospho-relay event (Håvarstein et al., 1996; Martin et al., 2010, 2012). Activated ComE promotes transcription of *comCDE* and other early competence genes (Ween et al., 1999). ComE-mediated transcription triggers robust competence among the cell population in a positive-feedback auto-regulatory loop. This response culminates in ComE dependent production of σ^X^ (Ween et al., 1999; Peterson et al., 2004). During competence, the σ^X^ – RNA polymerase (σ^X^ holo-enzyme) transcribes from the competence specific combox promoter, directly linking ComCDE quorum sensing to the expression of the transformation regulon (Peterson et al., 2004). Genes under control of the σ^X^ holo-enzyme include all genes that are required for DNA uptake and recombination.

Although σ^X^ is the master competence regulator required for late gene expression in pneumococcus, it is not sufficient for high transformation efficiencies. An additional ComE dependent early gene product, ComW, a 9.5 kDa DNA-binding protein (Luo et al., 2004; Inniss et al., 2019), is required for the strongest transformation phenotype possible. *sigX* is expressed independently of ComW (Luo et al., 2004; Piotrowski et al., 2009) and σ^X^ activity is dependent on the presence of ComW (Sung and Morrison, 2005). SigX’s requirement for ComW is supported by observations that mutants lacking ComW (Δ*comW*) transcribe late genes and transform at levels 10- and 10,000-fold below that of wild type cells, respectively (Tovpeko and Morrison, 2014). Furthermore, Δ*comW* cells have decreased σ^X^ levels (Sung and Morrison, 2005), and ComW and σ^X^ weakly interact in yeast-2-hybrid assays (Tovpeko et al., 2016), suggesting that they physically interact as protein partners in the cell. Interestingly, mutations to σ^A^ that presumably decrease its affinity for RNA polymerase (RNAP) alleviate the ComW requirement, indicating that ComW functions on or with the σ^X^ holo-enzyme. Beyond this, there is currently no evidence of a physical interaction between ComW and RNAP.

A structural model of ComW suggests that is structurally similar to known σ factors, and ComW binds to DNA, non-specifically (Inniss et al., 2019). DNA binding does not require residues ^73^RGFISC^78^, as demonstrated with the truncated variant, ComWΔ6, although ComWΔ6 producing cells transform at levels slightly below wild type cells (Inniss et al., 2019). Instead, the conserved ^38^LxYYLxR^44^ motif is important for DNA binding, and L42A and R44A mutations in the ^38^LxYYLxR^44^ motif decrease transformation efficiency in pneumococci (Inniss et al., 2019).

Although much is known about how σ^X^ functions in streptococcal species the details of how it facilitates combox promoter recognition and melting are not known. Moreover, how and why σ^X^-mediated transcription depends on ComW in unknown. Here we show that ComW stimulates transcription of competence genes, *in vitro*.

## Materials and Methods

### Bacterial Strains, Culture Media, and Plasmids

Bacterial strains and plasmids used are listed in Table 1. Plasmids pNLI60, pNLI115, and pNLI116 were synthesized by GenScript. *E. coli* strains DH5α and BL21De3 were hosts for plasmid isolation and protein expression, respectively. For plasmid introduction, *E. Coli* strains were chemically transformed according to (Mandel and Higa, 1970). *E. coli* strains were cultured in lysogeny broth (LB) (Bertani, 1951). LB was prepared from 5 g bacto tryptone (Difco), 5 g NaCl (Fisher) 2.5 g of yeast extract (Difco) in 1 L H_2_O, sterilized for 20 min at 121°C, and supplemented with appropriate antibiotics and 1.5% agar, as needed. Ampicillin was used at 100 μg/mL, for growth of *E. coli* strains. Antibiotics were purchased from Sigma-Aldrich.

**TABLE 1:** Bacterial strains, plasmids, and primers.

**Bacterial strains (strain, genotype)**		
*E. coli* DH5α	F- *φ80lacZ*Δ*M16* Δ(*lacZYA-argF*)U169 *recA1 endA1 hsdR17* (rk-mk +) *phoA supE44*λ*- thi-1 gyrA96 relA1*	
*E. coli* BL21(DE3)	F- *ompT hsdSB* (rB-mB-) *gal dcm* (DE3)	

**Plasmids (name, description)**		
pNLI60	pET22b+, Sp *comW*Δ*6*, C terminal fusion to V5His6 tag, under IPTG inducible promoter, Amp^*R*^	
pNLI94	pET22b+, Sp *sigX*, C-terminal fusion to V5His6 tag, under, IPTG inducible promoter, Amp^*R*^	
pNLI114	pET22b+, Sp *rpoD*, C-terminal fusion to V5His6 tag, under IPTG inducible promoter, Amp^*R*^	
pNLI115	pET22b+, partial Sp *comEA*, partial Sp *amiA*,	
pNLI116	pET22b+, partial Sp *ssbB*	

**Primers (name, sequence, gene target)**		
NL228	CGACGGTTGACAGCGATAGTTGC	*ssbB*
NL229	CAGATATGACCATTATGGCCAATCAACAG	*ssbB*
NL295	CATGAAAAAGGCCGAATCGTGACAAGAGTT	*comEA*
NL296	CCTGCAAATTCGTCTCTTTGACAGGTGTTT	*comEA*
NL299	CATTTTACTGTATGTCTTCCTAAACTCCAAAG	*amiA*
NL300	CATTTAACCCCTTTACGAATCTTATAAGTGTAG	*amiA*

*Sp, Streptococcus pneumoniae; V5H6, V5, and Hisx6 epitopes.*

### Expression and Purification of Pneumococcal Proteins From *E. coli*

Pneumococcal ComWΔ6 was expressed from pNLI60 and purified according to (Inniss et al., 2019). Pneumococcal σ^X^ and σ^A^ were expressed from pNLI94 and pNLI114, respectively. *E. coli* transformed with pNLI94 (SigX-V5H6, pI ∼8.42) or with pNLI114 (σ^A^-V5H6, pI ∼5.02) were cultured in 3 L of LB medium at 37°C, 200 rpm to an OD_600_ 0.5. To prepare a sample for SDS-PAGE, 2 mL of uninduced cells were collected and pelleted for 10 min at room temperature, and then boiled at 95°C for 2 min in a 3:1 mixture of *E. coli* resuspension buffer (50 mM Tris-HCl, pH 8.0, 500 mM NaCl) and Laemmli’s buffer (Bio Rad), respectively, then stored at 4°C. The large culture was induced for protein expression by addition of [1 mM]_f_ isopropyl β-D-1-thiogalactopyranoside (IPTG) (Gold Biotechnology). Induced cultures were incubated overnight at 20°C, 200 rpm. The next day, 2 mL of induced culture was prepped for analysis by SDS PAGE, and the large cultured collected at 5,000 rpm, 4°C, for 30 min. Cells were resuspended in 50 mL of *E. coli* resuspension buffer supplemented with 25 mM imidazole, 50 mM MgCl_2_, 100 μg/mL DNase, and a protease inhibitor tablet (Roche). The cell resuspension was lysed using an Emulsiflex-C3 (Avestin). The soluble fraction was separated by centrifugation at 30,000 × g for 30 min at 4°C.

For σ^X^ purification, the soluble fraction was discarded. The pellet was washed in 15 mL of cold, filtered 20 mM L-arginine, 1 mM EDTA, 5% glycerol (RGE buffer, final pH, 10.95) supplemented with 1 mM βME and 2% w/v sodium deoxycholate (NaDOC) (Sigma Aldrich) with a homogenizer, followed by sonication for 2 min at 65% amplitude (0.8/0.2, on/off cycles). The washed pellet was spun was 30,000 × g for 30 min at 4°C. Washing, sonication, and spinning were repeated. Then the pellet was resuspended and dissolved in 15 mL of RGE supplemented with 1 mM βME and 0.6% N-lauroyl sarcosine sodium salt (Sigma-Aldrich) at 4°C with slow stirring for up to 2 days. The dissolved inclusion body was collected by centrifugation at 30,000 × g for 30 min at 4°C. The supernatant was saved and dialyzed for 8 h at room temperature in 2 L of 25 mM Tris-HCl, pH 8.3, 500 mM NaCl, 30 mM L-arginine, 10% glycerol, and 5 mM βME (CB4), and then overnight at 4°C in 2 L of CB4. The soluble supernatant was passed through a 5 mL Ni-NTA resin (Thermo Fisher Scientific) on a column equilibrated with CB4 (no βME) via gravity flow. The column was washed with 500 mL of CB4 supplemented with 5 mM βME and 40 mM imidazole. SigX-V5H6 was eluted from the column with 10 mL of CB4 supplemented with 5 mM βME and 275 mM imidazole. Subsequently, the flow through was saved and concentrated using a Vivaspin 15R 2,000 MWCO column concentrator (Sartorius). The concentrated solution was applied to a pre-packed Superdex 200 column (GE) on an AKTA (Amersham Biosciences). SigX-V5H6 was eluted at 0.3 mL/min in CB4 supplemented with 1 mM βME and collected in 1 ml fractions for analysis on SDS-PAGE gels. The fractions were pooled and concentrated again using a VivaSpin column concentrator. Protein concentration was measured at 5.6 mg/mL using a Nanodrop. Purified SigX-V5H6 was diluted to 1.2 mg/mL in CB4 supplemented with 1 mM EDTA, 1 mM βME and 20% glycerol for storage at −20°C until use.

For purification of σ^A^, the soluble fraction was separated by centrifugation at 30,000 × g for 30 min at 4°C. The soluble supernatant was passed through a 5 mL Ni-NTA resin (Thermo Fisher Scientific) on a column equilibrated with Column Buffer (50 mM Tris-HCl, pH 8.0, 500 mM NaCl, 10% glycerol) via gravity. The column was washed with 500 mL of Column Buffer supplemented with 5 mM βME and 40 mM imidazole. σ^A^ was eluted from the column with 10 mL of column buffer supplemented with 5 mM βME and 275 mM imidazole. The eluate was concentrated using a Vivaspin 15R concentrator column into 50 mM Tris-HCl pH 8.0, 20 mM L-arginine, 500 mM NaCl, 1 mM EDTA, 10% glycerol, and 1 mM βME (DBR buffer). Protein concentration was measured as 1.85 mg/mL using a Nanodrop. SigA-V5H6 was diluted to 1 mg/mL in DBR supplemented with 20% glycerol and stored at −20°C until use.

### Amplification of Linear Templates for *in vitro* Transcription

Primers used in this study are listed in Table 1. Gene sequences for *amiA*, *comEA*, and *ssbB* were taken from the genome of *S. pneumoniae* R6 (NC_003098.1). Oligonucleotides were synthesized by IDT (Coralville, Iowa). Equal amounts (1 μM) of forward and reverse primers were used to amplify *amiA* (NL299/NL300), *comEA* (NL295/NL296), and *ssbB* (NL228/NL229) from 50 ng of CP2137 genomic DNA with 0.2 mM dNTP (Thermo Fisher Scientific) and 1 μL of Phire Hot Start DNA Polymerase II (Thermo Fisher Scientific) in 50 μL reactions in a thermocycler. PCR products were purified with Zymo kits, eluted in diethyl pyrocarbonate (DEPC) H_2_O and visualized on a 1% agarose gel prior to storage at −20°C.

### *In vitro* Transcription From DNA Templates

*In vitro* transcription from linear templates was carried out using 100 nM of *E. coli* RNAP obtained from NEB, pneumococcal σ^X^ and σ^A^ purified from *E. coli*, and ComWΔ6 purified from *E. coli*, when needed. To determine the minimum concentration of each σ-factor required to stimulate RNAP, initiation complexes were formed with 100 nM of *E. coli* RNAP, 20 nM of *ssbB* or *amiA*, and 0–200 nM of σ^X^ or σ^A^, in 50 mM Tris-acetate, 50 mM sodium-acetate, 12.5% glycerol, 0.25 mM EDTA, 15 mM magnesium-acetate, and 35 mM βME (4X TGA buffer). Transcription from plasmid templates was carried out using 0–400 nM of *E. coli* RNAP and 0–320 nM of σ^A^ or σ^X^. Solutions were incubated at 37°C for 15 min in a thermocycler, followed by addition of 1 μL of a master mix containing (120 nM) ATP, CTP, and GTP (Thermo Fisher Scientific) with ^α^ ^*P*32^UTP at 0.5 μCi/μL (3000 ci/mmol, Perkin Elmer). Runoff transcription was allowed to proceed for 6 min at 37°C in a thermocycler, followed by addition of 20 nM cold UTP (0.5 μL of a 200 nM stock) and a final elongation step at 37°C for 10 min in a final volume of 5.5 μL. Reactions were stopped by addition of one volume of formamide dye (80% formamide, 10 mM EDTA pH 8.0, 1 μg/mL xylene cyonal, and 1 μg/mL bromophenol blue) and incubation at 65°C for 2 min in a thermocycler. The samples were placed on ice until gel loading.

To measure transcription stimulation by ComWΔ6 on PCR templates, 100 nM of *E. coli* RNAP, 80 nM σ^X^ or σ^A^, 20 nM of *comEA*, *ssbB*, or *amiA*, and 0–160 nM of ComWΔ6 were mixed in 50 mM Tris-acetate, 50 mM sodium-acetate, 12.5% glycerol, 0.25 mM EDTA, 15 mM magnesium-acetate, and 35 mM βME (4X TGA buffer). Solutions were incubated at 37°C for 15 min in a thermocycler, followed by addition of 1 μL of a master mix containing (120 nM) ATP, CTP, and GTP (Thermo Fisher Scientific) with ^α^ ^*P*32^UTP at 0.5 μCi/μL (3000 ci/mmol, Perkin Elmer). Runoff transcription was allowed to proceed for 6 min at 37°C in a thermocycler, followed by addition of 20 nM cold UTP (0.5 μL of a 200 nM stock) and a final elongation step at 37°C for 10 min in a final volume of 5.5 μL. Reactions were stopped by addition of one volume of formamide dye (80% formamide, 10 mM EDTA pH 8.0, 1 μg/mL xylene cyonal, and 1 μg/mL bromophenol blue) and incubation at 65°C for 2 min in a thermocycler. The samples were placed on ice until gel loading.

To visualize mRNA transcripts, 2 μL of each reaction was run on a 20 × 20, 0.375 mm thick, 6% polyacrylamide urea gel at 20 watts for 45 min. Gels were dried for 40 min at 80°C and incubated on a phosphorimager and imaged on a Typhoon 3000 (GE). The expected mRNA transcript sizes were determined using the transcriptional start sites (TSS) for each gene as determined by Aprianto et al. (2018). Experimental transcripts were compared to markers generated by *in vitro* transcription using the MAXIscript T7 Transcription Kit (Invirtogen) and the RNA Century-Plus Marker Templates (Invitrogen).

## Results

### Pneumococcal σ-Factors Stimulate *Escherichia coli* Core RNA Polymerase, *in vitro*

*In vitro* transcription assays confirmed that σ^X^, in molar excess to RNAP, promotes transcription from combox promoters (Luo et al., 2003). However σ^X^-mediated late gene transcription and transformation requires ComW *in vivo* (Sung and Morrison, 2005). Previous work concluded that pneumococcal RNAP exists at ∼2000 molecules per cell, and that σ^X^ and ComW peak at ∼3000 and ∼500 molecules per cell, respectively (Luo and Morrison, 2003; Piotrowski, 2010). Although not confirmed, σ^X^ might have a lower affinity for RNAP than σ^A^, a trait shared among multiple alternative σ-factors (Gruber and Gross, 2003), so excess σ^X^ could increase the amount of σ^X^ holo-enzyme formation. Interestingly, levels of σ^A^ decrease 20–40% during the pneumococcal competence response (Luo and Morrison, 2003; Luo et al., 2003), and mutations to σ^A^, that presumably decrease its affinity for RNAP, can suppress Δ*comW* phenotypes (Tovpeko and Morrison, 2014; Tovpeko et al., 2016). These data hint that σ^X^ access to RNAP and/or competence promoters is an important step in efficient competence gene expression. In addition, we have shown that ComW, a likely σ^X^ binding partner (Tovpeko et al., 2016), interacts with DNA and this interaction is important for transformation (Inniss et al., 2019). We hypothesize that ComW functions at or near competence promoters to boost σ^X^ transcriptional activity. We tested this theory with *in vitro* transcription (IVT) assays.

Luo and Morrison used a ∼1.7 molar excess of σ^X^ to pneumococcal RNAP (RNAP_Sp_) in their IVT assays, making the number of molecules of σ^X^ higher than that of RNAP_Sp_ (roughly 5.12 × 10^12^ vs. 3.13 × 10^12^ molecules, respectively, and based on the average molar ratio of σ^X^:RNAP_Sp_ in cells as determined by Luo and Morrison, 2003). Thus, IVT from every competence template tested was successful and did not require ComW. As RNAP is conserved in prokaryotes (Murakami and Darst, 2003), we titrated pneumococcal σ^X^ and σ^A^ to determine the minimal amount of each σ-factor required to target 100 nM of *E. coli* RNAP (RNAP_Ecoli_) to the pneumococcal combox and housekeeping promoters, *in vitro*.

**FIGURE 1:**
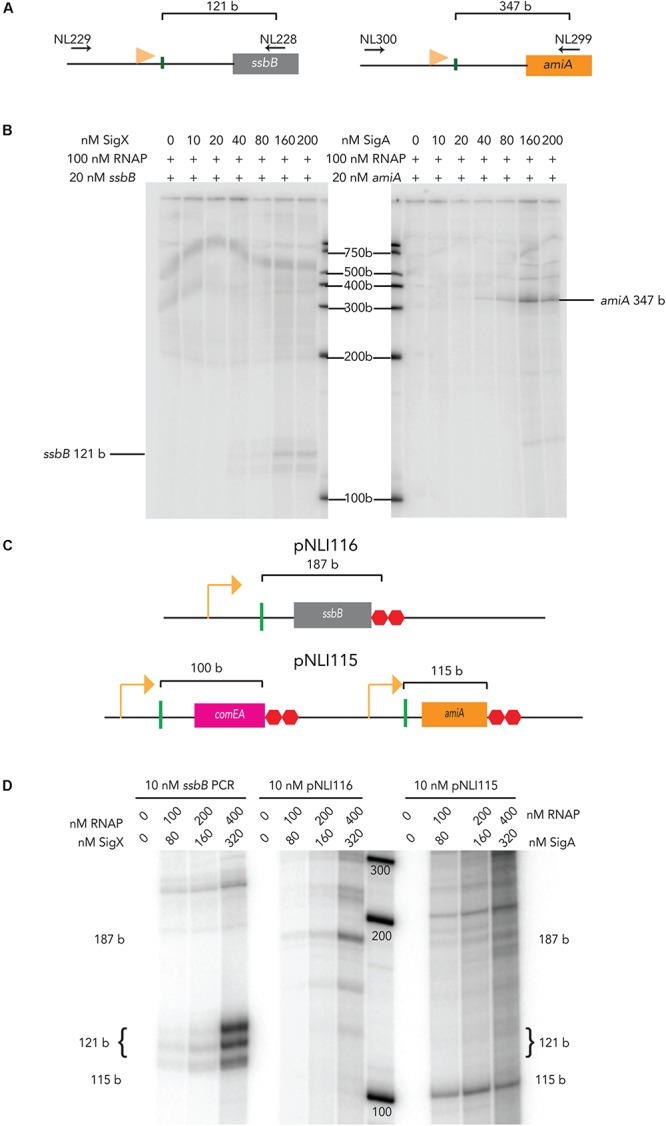
*E. coli* RNAP activation by pneumococcal sigma factors on linear and plasmid templates. **(A)** Schematic of *ssbB* (left) and *amiA* (right) PCR templates amplified from CP2137 genomic DNA using gene specific primers (their positions are indicated by the horizontal black arrows). The horizontal orange flags mark the positions of the combox promoter for *ssbB* and the Pribnow promoter box for *amiA*. The vertical green lines mark the transcription start sites for each gene, and the brackets indicate the size of the expected mRNA product (Aprianto et al., 2018). **(B)** The mRNA products from titration of pneumococcal σ^X^ (left blot) or σ^A^ with (right blot) with 100 nM of *E. coli* RNA polymerase and 20 nM of *ssbB* (left) or *amiA* (right) PCR templates. Pneumococcal σ-factors were titrated up to 200 nM. **(C)** (Top) Schematic of relevant region of plasmid pNLI116 containing pneumococcal combox promoter upstream of an *ssbB* gene fragment. (Bottom) Schematic of the relevant region of plasmid pNLI115, containing pneumococcal combox promoter upstream of *comEA*, followed the promoter upstream of pneumococcal *amiA*. The promoter upstream of each gene fragment is indicated by an orange arrow, and the vertical green lines mark the transcription start sites, with brackets that indicate the size of the expected mRNA product (Aprianto et al., 2018), and two red octagons indicate one copy of a pneumococcal termination signal (Aprianto et al., 2018) and a *ltR2* terminator. **(D)** mRNA products that result from *in vitro* transcription with pneumococcal σ^X^ or σ^A^ and *E. coli* RNA polymerase from 10 nM of PCR or plasmid template with *ssB* or *amiA* gene fragments, respectively. The sizes of the mRNA products produced are indicated on the side of the gel, and were estimate based on Aprianto et al. (2018).

The promoter regions of pneumococcal *ssbB* and *amiA* were PCR amplified for use in *in vitro* transcription assays (Figure 1A). At 40 nM σ^X^ and 100 nM RNAP_Ecoli_, a 121-base (b) mRNA product from 20 nM of an *ssbB* linear template was produced, although the amount of mRNA produced (Figure 1B). The amount of *ssbB* transcript produced increased as σ^X^ was titrated up to 160 nM. At 80 nM, there are approximately 1.93 × 10^11^ molecules of σ^X^ versus 2.41 × 10^11^ molecules of RNAP_Ecoli_ at 100 nM in the reaction. This molar ratio (80 nM σ^X^: 100 nM RNAP_Ecoli_) allowed for observable, yet sub-optimal transcription from 20 nM of linear *ssbB* DNA template containing a combox promoter, without ComW. In addition, 80 nM of σ^X^ + 100 nM RNAP_Ecoli_ did not produce an mRNA product when incubated with 20 nM of linear *amiA* template, a gene under control of the primary σ-factor, σ^A^ (not shown), demonstrating that σ^X^ transcription specifically initiates from the combox promoter.

In contrast at 20 nM, pneumococcal σ^A^ produced detectable levels of a 347-base mRNA product from the housekeeping promoter upstream of *amiA* when incubated with RNAP_Ecoli_ (Figure 1B). The amount of *amiA* transcript produced increased as σ^A^ was titrated up to 160 nM, with a detectable *amiA* transcript at 80 nM. Interestingly, we did observe production of an *ssbB* transcript with 80 nM of σ^A^ (not shown). This might result from an orphan -10 promoter region σ^A^ binding site that is present 29 bp downstream of the *ssbB* transcription start site, according to Aprianto et al. (2018). The nature of this mRNA product must be examined more thoroughly. Nonetheless, these results demonstrate that both pneumococcal σ-factors direct RNAP_Ecoli_ to pneumococcal promoters, *in vitro*. In addition, as it appeared that more σ^X^ was required to stimulate RNAP_Ecoli_ activity at combox promoters than σ^A^ at housekeeping promoters, these results might indicate that more σ^X^ is needed to stimulate RNAP_Sp_. As 80 nM of σ^X^ and σ^A^ are below saturating concentrations but still produced visible *ssbB* and *amiA* transcripts, respectively, we used 80 nM of each σ-factor in subsequent reactions.

To further explore *in vitro* transcription with pneumococcal σ factor, we tested transcription from plasmid templates. Supercoiling can affect bacterial gene transcription (Pruss and Drlica, 1989; Zhi et al., 2017), thus we asked whether or not our chimeric holo-enzymes could transcribe the *ssbB* and *amiA* genes from these plasmid templates. We designed two plasmids that carry pneumococcal promoters upstream of pneumococcal gene fragments, pNLI115 and pNLI116 (Figure 1C). pNLI116 contained only the combox promoter followed by an *ssbB* gene fragment, similar to our original PCR *ssbB* IVT template. pNLI115 contained the combox promoter region upstream of competence gene fragment *comEA*, and the housekeeping promoter region upstream of *amiA*, to serve as an internal control for promoter specific IVT from plasmid assays. After isolation from *E. coli*, each plasmid preparation contained predominantly supercoiled species, as observed on DNA agarose gels.

We titrated holo-enzymes into IVT reactions with 10 nM of either PCR DNA templates or plasmid templates. As expected, the amount of the 121 b mRNA transcript produced from linear DNA increased as the amount of σ^X^ holo-enzyme increased. Similar results were observed from pNLI116, as increasing amounts of a 187 b mRNA transcript was produced as the amount of σ^X^ holo-enzyme was titrated onto the plasmid (Figure 1D, left). In addition, a 115 b mRNA transcript was produced from pNLI115 when σ^A^-RNAP_*E. coli*_ complex was titrated onto the plasmid (Figure 1D, right). Together these data show that our chimeric holo-enzymes can be targeted to specific promoters on either linear or plasmid DNA templates, *in vitro.*

### ComWΔ6 Stimulates Transcription From σ^X^ Promoters, *in vitro*

**FIGURE 2:**
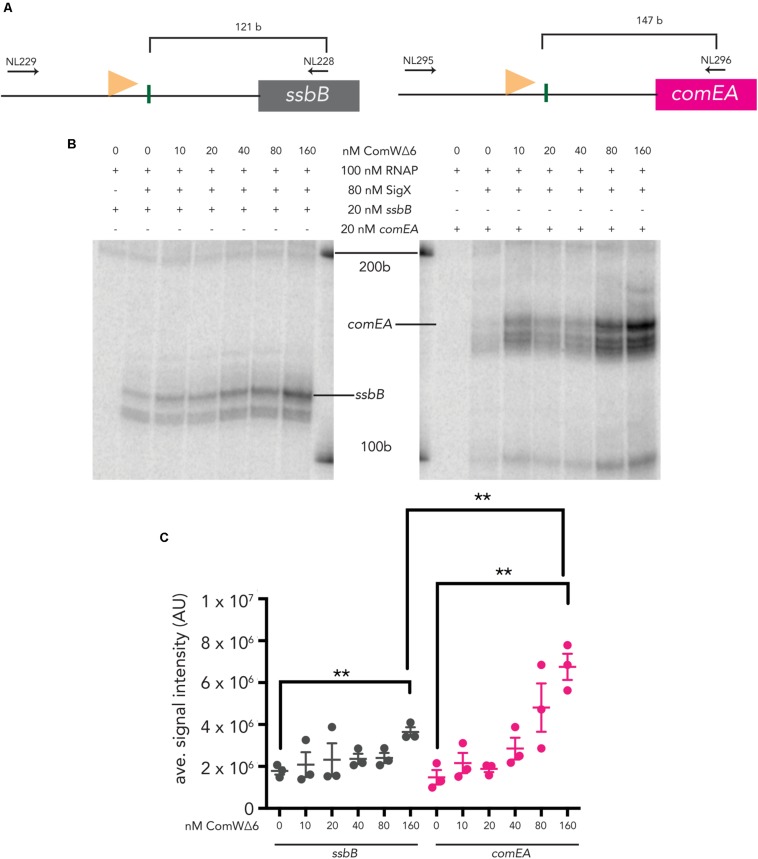
*In vitro* transcription from two pneumococcal late competence promoters using ComWΔ6. **(A)** A schematic of pneumococcal *ssbB* (left) and *comEA* (right) templates. A light orange flag represents each promoter and vertical green lines mark the transcription start sites. Horizontal arrows mark the positions of the primers used to amplify each template. **(B)** An image of *ssbB* (121 b, left) and *comEA* (147 b, right) mRNA products produced by σ^X^ holoenzymes with increasing amounts of ComWΔ6. The positions of the 100 b and 200 b standard bands are indicated. **(C)** Quantification of the signal intensities of mRNA transcripts from three *in vitro* transcription experiments. Asterisks mark statistically significant differences between samples with 0 nM ComWΔ6 and 160 nM ComWΔ6, and between samples with *ssbB* and *comEA* templates.

Both *ssbB* and *comEA* are induced by CSP and require ComW for robust transcription (Sung and Morrison, 2005). To determine the effect of ComW on transcription from combox promoters upstream of these two genes, we titrated ComWΔ6 into IVT reactions with σ^X^ holo-enzyme. Transcripts from both linear *ssbB* and *comEA* templates (Figure 2) were produced when ComWΔ6 was added to IVT reactions. Specifically, the σ^X^ holo-enzyme incubated with ComWΔ6 at 160 nM (2X molar excess of ComW to σ^X^) produced 2X as much mRNA signal from the *sbbB* template compared to reactions with 0 nM ComWΔ6, an increase that was statistically significant (Figure 2). Similarly, the σ^X^ holo-enzyme incubated with ComWΔ6 at 160 nM produced 4.5X as much mRNA signal from the *comEA* template compared to σ^X^ holo-enzyme with no ComWΔ6, also an increase that was statistically significantly (Figure 2). The 4.5X increase in *comEA* mRNA signal production versus the 2X increase in *ssbB* signal production also represented a statistically significant difference. These data demonstrate that ComW is required for robust transcription when the number of σ^X^ molecules is below the number of core RNAP molecules, *in vitro*. Furthermore, the difference in transcription stimulation suggests that the requirement for ComW at combox promoters differs. Lastly, as ComWΔ6 can stimulate σ^X^-mediate transcription, *in vitro*, it is possible that residues ^73^RGFISC^78^ are dispensable for transcription activation in pneumococci.

## Discussion

Anginosus and mitis group streptococci like the pneumococcus use the DNA binding protein, ComW, to regulate σ^X^-mediated transcription. However, previous data show that σ^X^ transcribes late genes in the absence of ComW, *in vitro* (Luo and Morrison, 2003). As we hypothesized that ComW is an active and important member of the competence-specific holo-enzyme, we examined how ComWΔ6 affected IVT from σ^X^ promoters. Remarkably, two σ^X^ targets, *ssbB* and *comEA* appeared to respond differently to the addition of ComWΔ6 to reactions; although transcription stimulation was observed for both genes, the level of stimulation appears greater for *comEA*. This is common in other naturally transformable species. For example, the *V. cholerae* competence regulator, QstR, binds upstream of some competence genes, but not others (Jaskólska et al., 2018), and the number of AT boxes that lie upstream of competence promoters in *B. subtilis* can differ from gene to gene, likely reflecting the difference in ComK activity at these promoters (Hamoen et al., 2003). Thus, at this moment, we cannot rule out that ComW is specific for transcription of some pneumococcal late competence genes, and might be dispensable for the transcription of others.

Interestingly, it appears that σ^A^ better stimulates *E. coli* RNAP than σ^X^. This might suggest that pneumococcal RNAP responds similarly to these two σ factors, and the specifics of pneumococcal σ – RNAP interactions must be determined. This could be another reason as to why σ^X^ – mediated transcription requires ComW in the pneumococcus. Furthermore, the σ^X^ – ComW pair appear similar to a two-part σ factor, σ^*O*^ – RsoA, identified in *B. subtilis* (Maclellan et al., 2009). In this system RsoA aids in σ^*O*^ – RNAP open complex formation to promote transcription of genes required during growth in acidic conditions. Like σ^*O*^, σ^X^ likely provides promoter recognition, and ComW, like RsoA, might aid in stabilizing the σX complex at the promoter (Maclellan et al., 2009). However, the specifics of a probable σX – ComW or ComW – RNAP interaction are yet to be determined.

**FIGURE 3:**
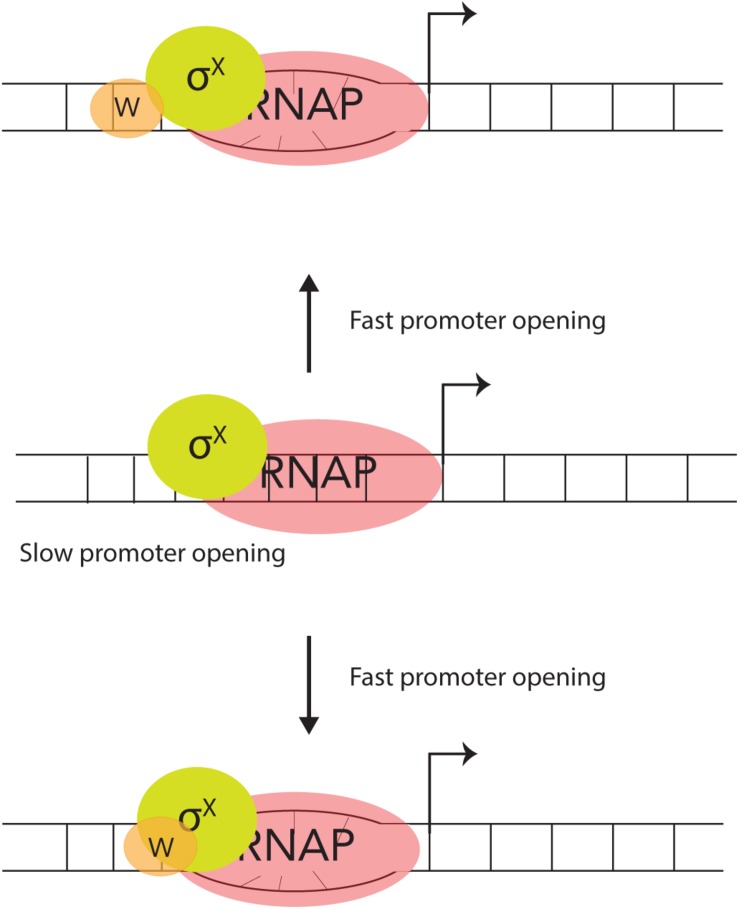
Two proposed mechanisms for ComW-dependent promoter melting. In the absence of ComW the pneumococcal σ^X^-holoenzyme can recognize and bind to combox containing DNA, but is slow to melt the promoter (middle). ComW is brought to combox promoters via interaction with σ^X^ (top) and/or RNAP (bottom). ComW uses its non-specific DNA binding function to aid in melting promoter DNA.

The DNA – binding protein, ComW does stimulate σ^X^ – mediated transcription, *in vitro*. Although more effort is required to fully work out the details, we posit that there are likely two possible roles for how ComW functions (Figure 3). (A) As ComW interacts non-specifically with DNA, it is likely brought to competence promoters by σ^X^, and increases promoter melting. (B) ComW might function as a component of a two-part σ factor with σ^X^ and RNAP as an active member of the holo-enzyme.

## Data Availability Statement

The datasets generated for this study are available on request to the corresponding author.

## Author Contributions

NI conceived, conducted experiments and wrote the manuscript. DM edited the manuscript.

## Conflict of Interest

The authors declare that the research was conducted in the absence of any commercial or financial relationships that could be construed as a potential conflict of interest.
